# Ampicillin/Sulbactam versus Cefuroxime as antimicrobial prophylaxis for cesarean delivery: a randomized study

**DOI:** 10.1186/1471-2334-10-341

**Published:** 2010-11-30

**Authors:** Eleftherios Ziogos, Sotirios Tsiodras, Ioannis Matalliotakis, Helen Giamarellou, Kyriaki Kanellakopoulou

**Affiliations:** 1 Department of Obtsterics and Gynecology, University General Hospital of Heraclion, Heracleion, Greece; 2 4th Academic Department of Internal Medicine, University of Athens Medical School, Athens, Greece

## Abstract

**Background:**

The efficacy and safety of a single dose of ampicillin/sulbactam compared to a single dose of cefuroxime at cord clamp for prevention of post-cesarean infectious morbidity has not been assessed.

**Methods:**

Women scheduled for cesarean delivery were randomized to receive a single dose of either 3 g of ampicillin-sulbactam or 1.5 g of cefuroxime intravenously, after umbilical cord clamping. An evaluation for development of postoperative infections and risk factor analysis was performed.

**Results:**

One hundred and seventy-six patients (median age 28 yrs, IQR: 24-32) were enrolled in the study during the period July 2004 - July 2005. Eighty-five (48.3%) received cefuroxime prophylaxis and 91 (51.7%) ampicillin/sulbactam. Postoperative infection developed in 5 of 86 (5.9%) patients that received cefuroxime compared to 8 of 91 (8.8%) patients that received ampicillin/sulbactam (p = 0.6). In univariate analyses 6 or more vaginal examinations prior to the operation (p = 0.004), membrane rupture for more than 6 hours (p = 0.08) and blood loss greater than 500 ml (p = 0.018) were associated with developing a postoperative surgical site infection (SSI). In logistic regression having 6 or more vaginal examinations was the most significant risk factor for a postoperative SSI (OR 6.8, 95% CI: 1.4-33.4, p = 0.019). Regular prenatal follow-up was associated with a protective effect (OR 0.04, 95% CI: 0.005-0.36, p = 0.004).

**Conclusions:**

Ampicillin/sulbactam was as safe and effective as cefuroxime when administered for the prevention of infections following cesarean delivery.

**Trial registration:**

Clinicaltrials.gov identifier: NCT01138852

## Background

The majority of obstetrical interventions involves some degree of bacterial contamination. Thus they are classified as "clean-contaminated" even when the patient has no preoperative symptoms of active infection [1]. Cesarean delivery is considered a clean-contaminated procedure when scheduled cesarean delivery without labor and/or ruptured membranes occurs and, contaminated when emergency cesarean delivery with labor and/or ruptured membranes occurs. Several studies have shown the beneficial effect of peri-operative antimicrobial prophylaxis in preventing post-surgical infection after cesarean delivery [2]. Single dose prophylaxis appears to be an excellent regimen compared to multiple day regimens independent of urgency of procedure [2-6]. Most surgical - site infections (SSI) after cesarean delivery, are soft tissue infections caused by organisms originating in the lower genital tract such as gram negative microorganisms and anaerobes [7-9]. Some authors argue that the most effective regimen has not been established yet [10]. For example single dose cephalosporins have been widely used for antimicrobial prophylaxis during cesarean delivery [11-13]. However in one study it was shown that a broader combination of cefazolin and metronidazole provided better efficacy with regards to post-operative infectious diseases morbidity and duration of hospitalization when compared with cefazolin alone [10]. The antimicrobial combination of ampicillin-sulbactam has broader spectrum of activity compared to first and second generation cephalosporins [14]. This activity includes enterococci and anaerobes and in a recent randomized trial it was shown to be superior than cehalosporins in perioperative chemoprophylaxis in biliary surgery [15]. Moreover, in an obstetrical study it fared better than ampicillin alone in preventing post-cesarean infection in women that had ruptured membranes [16].

In the current study, the main goal was to evaluate the efficacy and safety of a single dose of ampicillin/sulbactam compared to a single dose of cefuroxime at cord clamp for prevention of post-cesarean infectious morbidity. The main hypothesis was that ampicillin-sulbactam will result in fewer post-cesarean infections.

## Methods

The investigation was designed to evaluate the efficacy and safety of a single dose of ampicillin/sulbactam 3 g compared to a single dose of cefuroxime 1.5 g in preventing postoperative morbidity. The primary outcome was development of an infection either at the surgical site or elsewhere e.g. urinary tract infection. A prospective randomized controlled study was performed from July 2004 to December 2008 in one major tertiary care hospital, Nikaia's Regional General Hospital "Agios Panteleimon", in Athens Greece. All patients undergoing a cesarean delivery were eligible. Using a random-number generator (STATS version 1.1, 1998 program; Decision-Analyst Inc, Arlington, Tex), patients were randomly assigned to receive either 1.5 g of cefuroxime, or 3 g of ampicillin/sulbactam intravenously after the time the umbilical cord was clamped. The generation of the allocation sequence, the enrollment and the assignment of participants to their groups was performed by a physician dedicated to the study. The sequence was obtained using a central telephone number and it was concealed until interventions were assigned. Participants were enrolled sequentially and were blinded to the intervention, however the physician administering the intervention and assessing the outcomes was not. Patients with known hypersensivity to penicillin, cephalosporins, those who required concomitant antibiotic therapy or had received antibiotics during the 72 hours immediately preceding their enrollment, were excluded. Prior to the enrollment in the study, the medical history was taken and a physical examination was performed. Then the procedure was explained and discussed with each patient and written informed consent was obtained when a decision to proceed to a cesarean delivery was made. Preoperative vital signs including blood pressure, pulse rate, and temperature were also recorded. Blood samples were taken for complete blood counts with differential and blood chemistries. Concerning patients with diabetes mellitus, glycemic control was established preoperatively by close monitoring and insulin drips, if appropriate. Data on parity, indication of cesarean delivery, number of vaginal examinations, premature ruptures of membranes, duration of surgery, blood loss, type of anesthesia, BMI and American Society of Anesthesiologists (ASA) score were carefully recorded.

The patients were under weekly clinical and laboratory postoperative monitoring for infection development during a 30- day period. All of them were reminded of the appointment via telephone, and a structured clinical protocol was used. The following information was recorded daily during the postoperative hospitalization and the follow-up period: the patient's vital signs, clinical signs and symptoms of wound infection (such as induration, heat, erythema, pain and drainage from the wound), and signs and symptoms of infections of other sites. Postoperative superficial or deep incision soft tissue SSI and intraabdominal abscess were defined according to published criteria [1]. Endometritis was defined as the presence of fever (38°C or higher) in association with one or more of the following: uterine tenderness, foul smelling lochia, and leucocytosis of >12.000 after exclusion other site of infection that developed within the first 5 days after the delivery [17]. Clinical sense and caution was exercised not to include normal leukocytosis that can be seen during pregnancy and immediately post-partum. Endometritis qualified also as organ SSI. Presence of fluid collection with local signs of inflammation with or without fever, with or without leukocytosis, and with negative culture results was defined as a sterile wound collection. Specimens for both aerobic and anaerobic bacteriologic culture were obtained in case of a postoperative infection and from all wounds. The prophylaxis was considered to have failed if a postoperative infection occurred. Mothers whose postpartum fever was clearly associated with other known causes such as deep vein thrombosis were excluded. The study was conducted in accordance with the Declaration of Helsinki, was approved by the institutional scientific review board and the ethics committee of the Nikaia's Regional General Hospital "Agios Panteleimon"and all patients participating in the study signed an informed written consent. The study was registered in the National Institutes of Health clinical trials registry (Clinicaltrials.gov identifier: NCT01138852).

### Statistical analysis

Data are reported as mean (SD), rates or odds ratio (OR) with 95% confidence interval. We used a paired-sample t test for normally distributed data and a Wilcoxon signed rank test otherwise. Baseline characteristics were compared between the two groups for each of the study medication using non-parametric, independent-sample tests (Kruskal-Wallis and Wilcoxon rank) for continuous data and chi-square Fisher exact tests for categorical data. The drug efficacy was accessed by development of an infection either at the surgical site or elsewhere e.g. urinary tract infection. The association between known risk factors for infection e.g. comorbidities, ASA score, obesity (Body Mass Index ≥ 25 kg/m^2^, type (elective vs. non - elective) and duration of operation, blood loss, presence of bacterial vaginosis, preoperative vaginal examinations (also dichotomized as 6 or more), premature rupture of membranes (also dichotomized as 6 hours or more) and the development of any postoperative infection or SSI was examined by univariate analysis. Backward stepwise logistic regression analysis was then conducted to determine independent correlates of post-operative infection or SSI. Variables with P ≤ 0.10 in the univariate analysis were considered for inclusion in the multivariate analysis. Significance was set at p < 0.05. It was estimated according to published literature [2] that for an expected reduction of post-surgical infection between patients not exposed and exposed to ampicillin-sulbactam from approximately 15% [2] to 1.5%, a power of 80%, and a two-tailed alpha of 0.05, 78 patients would be required for each arm of the study. All statistical tests used were two-sided. SPSS version 10.0 for Windows software (SPSS, Inc., Chicago, IL) was used for data analysis.

## Results

**Figure 1:**
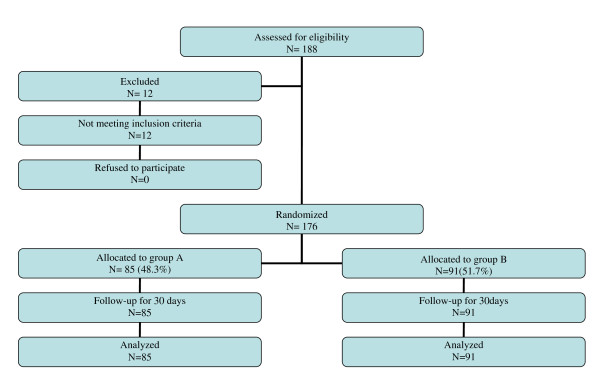
**Flow diagram of the study according to the Consort statement**.

**Figure 2:**
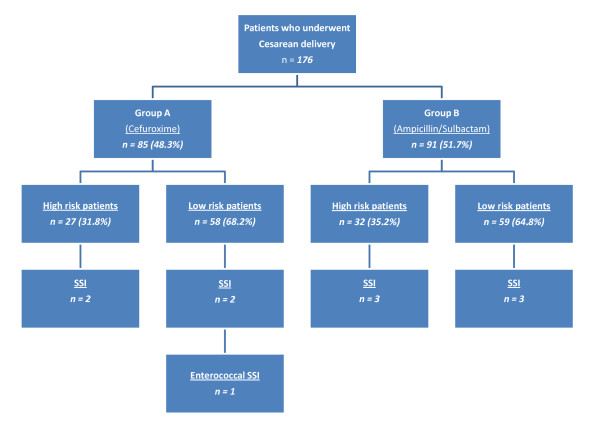
**A schematic diagram of the study according to prophylaxis group, operative risk (high versus low), type of surgery (elective, emergency cesarean delivery) and development of Surgical Site Infection (SSI)**.

A flow diagram of the study according to the Consort statement is depicted in Figure 1. In total 188 patients were assessed for eligibility and 176 were included in the study from July 2004 to July 2005. The only reason for exclusion was reported allergy to beta-lactam antibiotics. The main characteristics of the patients who participated in the study with respect to the prophylaxis group are presented in tables 1 and 2. Among the 176 patients [median age 28 yrs old (IQR: 24-32)] completing the study, 85 (48.3%) received cefuroxime and 91 (51.7%) ampicillin/sulbactam. There were no statistically significant differences in outcome parameters between the two treatment groups. Postoperative infections developed in 13 (7.4%) patients; five out of 85 (5.9%) patients that received cefuroxime and 8 out of 91 (8.8%) patients that received ampicillin-sulbactam (p = 0.6). More specifically 10 cases (5.7) of postoperative SSI developed (Figure 2), 6 in the ampicillin/sulbactam group and 4 in the cefuroxime group (p = 0.7). Five qualified as superficial incisional SSIs and 5 as organ/space SSIs (4 cases of post-partum endometritis and one of intrabdominal collection). Two SSIs were associated with a positive wound culture for *Staphylococcus aureus*, one with *Stapylococcus epidermidis *(deep infection with intra-abdominal collection), one with *Proteus mirabilis*, one with *Enterobacter aerogenes *and one with *Enterococcus faecalis*. The superficial incisional SSI that was attributed to *Enterococcus *spp developed in a patient that received prophylaxis with cefuroxime. Four cases of endometritis were observed, 2 in each group. All cases of endometritis were associated with the isolation of Gram negative aerobic bacteria [2 cases with *E. coli *(one had a concomitant urinary tract infection with the same pathogen) and one case each where *Citrobacter *spp and *Enterobacter cloacae *were isolated respectively]. Finally, another urinary tract infection developed and *E. coli *was cultured again. All pathogens except for the enterococcal species were susceptible to the 2 prophylaxis regimens used. No significant differences in risk factors for infection were identified between the two groups.

**Table 1:** Demographics and other characteristics of the population studied

Variable	Total group n = 176	Cefuroxime n = 85 (48.3%)	Ampicillin/Sulbactam n = 91 (51.7%)	P - value
Age, median (IQR)	28 (24-32)	27 (23.5-31)	28 (24-32)	0.7

Multiparity, n (%)	98 (55.7)	48 (56.5)	50 (54.9)	0.9

BMI ≤24 kg/m^2^, n (%)	39 (22.2)	18 (21.2)	21 (23.1)	0.9

BMI ≥ 30 kg/m^2^, n (%)	76 (43.2)	38 (44.7)	38 (41.8)	0.8

Diabetes mellitus, n (%)	7 (4)	3 (3.5)	4 (4.4)	1

Hypertension (including pre-eclampsia)/other cardiovascular condition, n (%)	36 (20.5)	16 (18.8)	20 (22)	0.7

Chronic respiratory disease, n (%)	1 (0.6)	1 (1.2)	0 (0)	0.5

Corticosteroid use, n (%)	2 (1.1)	2 (2.4)	0 (0)	0.2

Creatinine in mg/dL, median (IQR)	0.6 (0.5-0.7)	0.6 (0.5-0.7)	0.6 (0.5-0.7)	0.6

ASA ≥ 2, n (%)	59 (33.5)	27 (31.8)	32 (35.2)	0.7

Regular prenatal f/u, n (%)	167 (94.9)	83 (97.6)	84 (94.4)	0.4

Vaginal examinations, n (%)	96 (54.5)	45 (52.9)	51 (56)	0.8

Vaginal examinations ≥ 6, n (%)	23 (13.1)	11 (12.9)	12 (13.2)	1

Ruptured membranes, n (%)	54 (30.7)	24 (28.2)	30 (33)	0.5

Ruptured membranes ≥ 6 hours, n (%)	9 (5.1)	3 (3.5)	6 (6.6)	0.5

Non-elective emergency cesarean, n (%)	87 (49.4)	42 (49.4)	45 (49.5)	1

General anesthesia	110 (62.5)	50 (58.8)	60 (65.9)	0.4

Duration of surgery >60 minutes, n (%)	111 (63.1)	53 (62.4)	58 (63.7)	0.9

Blood loss >500 ml, n (%)	74 (42)	37 (43.5)	37 (40.7)	0.8

All post-op infections, n (%)	13 (7.4)	5 (5.9)	8 (8.8)	0.6

Post-cesarean endometritis, n (%)	4 (2.3)	2 (2.4)	2 (2.2)	1

Post-cesarean SSI (includes endometritis), n (%)	10 (5.7)	4 (4.7)	6 (6.6)	0.7

Duration of hospitalization in days, median (IQR)	4 (4-4)	4 (4-5)	4 (4-4)	0.09

Duration of hospitalization post-operatively in days, median (IQR)	2 (2-2)	2 (2-2)	2 (2-2)	0.04

BMI: Body mass index, ASA: American society of Anesthesiologists score, Post-op: postoperative, IQR: Interquartile range

**Table 2:** Variables studied in univariate analysis and their association with the development of any postoperative infection (n = 13) and with development of post-operative SSI (n = 10).

Variable	Post-op. Infection n = 13 (7.4)	No Infection n = 163 (92.6)	P	Post-op. SSI n = 10 (5.7)	No SSI n = 166 (94.3)	P
Age, median (IQR)	27 (24-32)	28 (24-32)	***1***	28 (23-32)	28 (24-32)	***1***

Multiparity, n (%)	7 (53.8)	91 (55.8)	***1***	6 (60)	92 (55.4)	

BMI >25 kg/m^2^, n (%)	13 (100)	124 (76.1)	***0.08***	10 (100)	127 (76.5)	***0.12***

BMI ≥ 30 kg/m^2^, n (%)	7 (53.8)	69 (42.3)	***0.6***	5 (5)	71 (42.8)	***0.7***

Diabetes mellitus, n (%)	0 (0)	7 (4.3)	***1***	0 (0)	7 (4.2)	***1***

Hypertension/other CVD, n (%)	0 (0)	36 (22.1)	***0.07***	0 (0)	36 (21.7)	***0.2***

Chronic respiratory disease, n (%)	0 (0)	1 (0.6)	***0.9***	0 (0)	1 (0.6)	***1***

Corticosteroid use, n (%)	0 (0)	2 (1.2)	***1***	0 (0)	2 (1.2)	***1***

Creatinine in mg/dL, median (IQR)	0.6 (0.5-0.85)	0.6 (0.5-0.7)	***0.2***	0.7 (0.5-0.8)	0.6 (0.5-0.7)	***0.2***

ASA ≥ 2, n (%)	7 (53.8)	52 (31.9)	***0.1***	5 (50)	54 (32.5)	***0.3***

Regular prenatal f/u, n (%)	11 (84.6)	156 (96.9)	***0.09***	8 (80)	159 (97)	0.05*

Vaginal examinations >1, n (%)	9 (69.2)	87 (53.4)	***0.4***	8 (80)	88 (53)	***0.11***

Vaginal examinations ≥ 6, n (%)	6 (46.2)	17 (10.4)	0.002*	5 (50)	18 (10.8)	0.004*

Ruptured membranes, n (%)	7 (53.8)	47 (28.8)	***0.1***	6 (60)	48 (28.9)	***0.07***

Ruptured membranes ≥ 6 hours, n (%)	2 (15.4)	7 (4.3)	***0.1***	2 (20)	7 (4.2)	***0.08***

Non-elective cesarean, n (%)	9 (69.2)	78 (47.9)	***0.2***	8 (80)	79 (47.6)	***0.056***

Type of anesthesia (general vs. regional), n of general (%)	5 (38.5)	105 (64.4)	***0.08***	2 (20)	108 (65.1)	0.006*

Duration of surgery >60 minutes, n (%)	10 (76.9)	101 (62)	***0.4***	7 (70)	104 (62.7)	***0.7***

Blood loss >500 ml, n (%)	10 (76.9)	64 (39.3)	0.016*	8 (80)	66 (39.8)	0.018*

Duration of hospitalization in days, median (IQR)	5 (4-8)	4 (4-4)	<0.001*	5 (4-8)	4 (4-4)	<0.001*

*: Significant associations p < 0.05. ASA: American society of Anesthesiologists score, BMI: Body mass index, CVD: Cardiovascular disease, Post-op: postoperative, IQR: Interquartile range

Variables that were associated with any infection or SSI during univariate analysis are shown in table 2. For SSI univariate analysis disclosed as important risk factors, ruptured membranes (OR 3.7, 95% CI: 0.99-13.7, p = 0.07), 6 or more vaginal examinations prior to the cesarean (OR 8.2, 95% CI: 2.2-31.2, p = 0.004), emergency cesarean (OR 4.4, 95% CI: 0.91-21.4, p = 0.056), and blood loss greater than 500 ml (OR 6.1, 95% CI: 1.2-29.4, p = 0.018). Regular prenatal follow-up (OR 0.13, 95% CI: 0.02-0.75, p = 0.05) and the performance of general anesthesia appeared to be protective (OR 0.13, 95% CI: 0.03-0.65, p = 0.06). For SSI multivariate analysis disclosed six or more vaginal examinations performed prior to the cesarean delivery as the most significant risk factor for a postoperative SSI (OR 6.8, 95% CI: 1.4-33.4, p = 0.019) and regular prenatal follow-up to be associated with a protective effect (OR 0.04, 95% CI: 0.005-0.36, p = 0.004). Regional anesthesia approached but did not reach statistical significance for an SSI development (OR 5.6, 95% CI: 0.9-36, p = 0.07). For development of any infection similar results to the SSI ones were obtained in univariate and multivariate analysis. In multivariate analyses having six or more vaginal examinations performed prior to the cesarean delivery was the most significant risk factor for any postoperative infection (OR 6.9, 95% CI: 1.8-26.1, p = 0.005) whereas regular prenatal follow-up was associated with a protective effect (OR 0.09, 95% CI: 0.01-0.6, p = 0.01). In the same analysis blood loss of greater than 500 ml approached but did not reach significance (OR 3.6, 95% CI: 0.9-15, p = 0.077).

Finally, patients that developed an infection had a lengthier hospital stay [median of 5 (IQR: 4 - 8) vs. 4 days (IQR: 4 - 4), Mann - Whitney, p < 0.001). All patients with an infection responded well to subsequent antibiotics. No adverse drug reactions were reported.

## Discussion

In this randomized trial, we report the efficacy of a single peri-operative dose of ampicillin-sulbactam or cefuroxime as chemoprophylaxis in patients undergoing cesarean delivery. Both cefuroxime and ampicillin-sulbactam prevented the occurrence of postoperative infections overall and SSI in specific. Both regimens were well tolerated and no side-effects were noted. The most significant risk factors for postoperative infection were 6 or more vaginal exams prior to the cesarean, blood loss of greater than 500 ml during the operation and inadequate prenatal follow-up. Patients with SSI had a lengthier hospital stay but all did well during follow-up.

Cephalosporins have been widely used as prophylaxis in obstetrics and gynecological surgery. However they are not active against *Enterococcus *spp [18]. Ampicillin-sulbactam has a wider antimicrobial spectrum than cephalosporins that includes enterococci. The use of a single dose of ampicillin/sulbactam as a prophylactic agent to prevent infection following cesarean delivery in both non-select and selct populations has been reported in the past [16,19,20]. One study has looked at the comparison between ampicillin-sulbactam and cefotetan (a cephalosporin with anaerobic activity frequently used in obstetrics and gynecology) in 170 patients and did not identified significant differences in rates of postoperative endometritis and urinary tract infections [19]. To the best of our knowledge no direct comparison of ampicillin-sulbactam and cefuroxime has been performed to date regarding their use as chemoprophylaxis. We elected to compare the two regimens assuming a benefit for the agent with the wider spectrum. In support of our hypothesis, enterococci are among the most frequent pathogens implicated in cases of post-partum emdometritis after cephalosporin prophylaxis [21-23]. Moreover a comparison of chemoprophylaxis with ampicillin-sulbactam versus ampicillin alone has been previously reported [16] and the combination fared better than the single agent in the group of women with ruptured membranes [16]. However, our study did not establish any superiority for the ampicillin-sulbactam regimen over cefuroxime. The likely reason is that we only observed one enterococcal post-operative infection out of 13 (for a rate of 7.7%) and no anaerobic infections. Based on calculation derived from our study figures, if all our patients were to develop a susceptible enterococcal post-operative infection we would have needed 34 patients developing such an infection to see 1 case of enterococcal infection prevented. Nevertheless other factors may be operative as well such as characteristics of the populations examined. The presented study highlights the importance of the appropriate use of a simple peri-operative prophylaxis regimen in patients undergoing cesarean section. The alternative regimen i.e. ampicillin-sulbactam presented in this report was associated with essentially negligible drug related toxicity and in addition it may be associated with reduced selection of pathogens resistant to cephalosporins such as enterococci. The one enterococcal SSI was noted in the cefuroxime treated patient. Moreover we had no occurrence of infection due to a resistant pathogen in our ampicillin-sulbactam treated group. On the other hand a wider spectrum antimicrobial such as ampicillin-sulbactam was not superior to a more narrow spectrum cephalosporin. In accordance a meta-analysis showed similar efficacy between ampicillin (alone without the combination with sulbactam) and first or second or even third generation cephalosporins in the prevention of endometritis [24]. More research is necessary as newer reports suggest that the use of extended-spectrum regimens (involving azithromycin or metronidazole) after cord clamp may reduce post-cesarean maternal infection by up to 50% [25]. Nevertheless the authors believe that simple narrow spectrum antimicrobials should continue to be used for perioperative prophylaxis (especially in low-risk patients) and should be integrated in an appropriate antimicrobial stewardship program There does not appear to be added benefit in utilizing a more broad spectrum and the authors believe that simple narrow spectrum antimicrobials (especially in low risk subjects) should continue to be used for perioperative prophylaxis and should be integrated in an appropriate antimicrobial stewardship program. On the other hand prophylaxis regimens should assist in reducing health care costs without an adverse effect in the quality of the care provided to the patient. The ampicillin-sulbactam regimen used in our study costs 5,07 Euros per patient in our country and was more costly than the cefuroxime regimen that costs 2,38 Euros per patient.

The current study confirms the well accepted beneficial role of antimicrobial prophylaxis for any woman undergoing elective or emergency cesarean delivery regardless of the presence or absence of specific risk factors. The operation is known to carry a 5- to 20-fold greater risk of infection than normal vaginal delivery [9,26]. The potential bacterial pathogens may contaminate the endometrial cavity at the time of cesarean delivery. During a cesarean delivery, the uterine incision, pelvic peritoneum and abdominal wound can become contaminated. Endometritis which is the most common infectious complication after delivery, is more frequent and severe after cesarean delivery [27,28]. Wound infection occurs in a large percentage of women undergoing a cesarean delivery without antibacterial prophylaxis [9,29]. Since the early studies [30] extensive research has examined the role of antimicrobial prophylaxis in preventing post-operative infections and especially endometritis. The benefit of prophylaxis is most clearly seen in those at increased risk for infection, for example, those in active labors and with ruptured membranes [3,26]. In a recent report, the risk of SSI increased 2.58 fold (95%CI, 1.3-5.1) in the absence of prophylaxis in a population of 765 women [29]. In a large meta-analysis it was shown that the use of antibiotic prophylaxis led to a reduction of up to 75% of cases of post-cesarean delivery endometritis and a decrease in wound infections [2]. This observation was independent of whether an elective or non-elective procedure was performed and independent of the antimicrobial regimen used [2]. The relative risk of endometritis reduction for the 11,937 patients analyzed was 0.39 (95% CI 0.31 to 0.43) [2]. Accordingly our study showed a rate of 7.4% for overall post-operative infections and a rate of 3.4% for post-operative SSI for the entire population examined.

Several risk factors for the development of a post-operative infection were identified in our study. Ruptured membranes, greater ASA score, having 6 or more vaginal exams performed prior to the cesarean, blood loss of greater than 500 ml during the operation and inadequate prenatal follow-up were the most important ones. Our findings concur with published literature on the subject [29,31]. Regarding prenatal follow-up it is possible that women with regular exams had conditions such as bacterial vaginosis that are predisposing factors for ruptured membranes, premature labor and post-partum infection. Of interest is the fact that previous research has identified an increased number of vaginal examinations and increased length of internal monitoring as risk factors for an enterococcus associated post-cesarean endometritis [21]. We were not able to explain the finding of regional anesthesia correlating with a higher risk for an SSI and this finding needs to be further explored. Physicians should identify those patients at highest risk for developing postoperative infections and maintain a close follow-up for the weeks following the cesarean delivery. If symptoms and signs of infection develop appropriate diagnostic testing should be performed and every effort made to identify an offending pathogen. Then appropriate antimicrobial therapy will be prescribed according to susceptibility testing. Gram negative flora predominated in the infections observed in our cohort.

Limitations of our study include the lack of quantitative microbiological data from women prior to the cesarean delivery as well as after the operation (except for qualitative data in cases of infection). This knowledge would have allowed a much better understanding of the role of the pathogen (and the prescribed antimicrobial prophylaxis regimen) in cesarean deliveries. For example, presence of a microorganism in the genital tract besides infection can also be found in instances of colonization creating difficulty in clarifying the exact pathogenetic role in instances of infection. The monomicrobial nature of the culture results in all cases of infection in our study, may have limited such a bias. Furthermore, we had a significant number of emergency procedures due to the nature of our institution which is a tertiary care center with several referrals from suburban and regional hospitals. This may have created a selection bias for patients at a higher risk for infection. In fact over 30% of the population studied had a high ASA score. Another limitation is that the physician assessing outcomes was not blinded to the group assignments due to technical reasons and his participation as an assistant in the vast majority of the operations performed. Since the indicators of infection can be subjective it is possible that the observations may have been influenced by knowledge of the treatment group. However, accurate recording of several variables relating to the operation per se was achieved due to this fact. A third limitation is that we could not account for any residual confounding in our risk factor associations. A final limitation is the fact that the prophylaxis regimen was administered right after the time the umbilical cord was clamped according to standard obstetrical practice. However, a very recent position statement was published by The American College of Obstetricians and Gynecologists supporting a change in the current practice and recommending that the prophylaxis should be given 1 hour prior to the operation. Since our study was performed in the past we could not adhere to this guideline which will be implemented in the near future.

## Conclusions

In conclusion, we report for the first time in the literature a randomized comparison of ampicillin-sulbactam vs. cefuroxime as single dose prophylaxis in cesarean delivery. Our study demonstrated equal efficacy of the two regimens regarding prevention of postoperative infections after cesarean delivery. Both antibiotics were safe and well tolerated with no unusual or unexpected events.

## Competing interests

The authors declare that they have no competing interests.

## Authors' contributions

EZ, KK, HG, conceived and designed the study, EZ performed the study, ST did the analysis and together with EZ prepared the first draft, all authors read, critically reviewed and approved the final draft of this manuscript.

## Pre-publication history

The pre-publication history for this paper can be accessed here:

http://www.biomedcentral.com/1471-2334/10/341/prepub
